# Strain Typing Methods and Molecular Epidemiology of *Pneumocystis* Pneumonia

**DOI:** 10.3201/eid1010.030981

**Published:** 2004-10

**Authors:** Charles Ben Beard, Patricia Roux, Gilles Nevez, Philippe M. Hauser, Joseph A. Kovacs, Thomas R. Unnasch, Bettina Lundgren

**Affiliations:** * Centers for Disease Control and Prevention, Fort Collins, Colorado, USA;; † Saint-Antoine University Hospital, Paris, France;; ‡ University of Picardy, Amiens, France;; § University Hospital of Lausanne, Lausanne, Switzerland;; ¶ National Institutes of Health, Bethesda, Maryland, USA;; # University of Alabama at Birmingham, Birmingham, Alabama, USA;; ** Hvidovre Hospital, Hvidovre, Denmark

**Keywords:** Pneumocystis, PCP, molecular epidemiology, typing methods, perspective

## Abstract

*Pneumocystis* pneumonia (PCP) caused by the opportunistic fungal agent *Pneumocystis jirovecii* (formerly *P. carinii*) continues to cause illness and death in HIV-infected patients. In the absence of a culture system to isolate and maintain live organisms, efforts to type and characterize the organism have relied on polymerase chain reaction–based approaches. Studies using these methods have improved understanding of PCP epidemiology, shedding light on sources of infection, transmission patterns, and potential emergence of antimicrobial resistance. One concern, however, is the lack of guidance regarding the appropriateness of different methods and standardization of these methods, which would facilitate comparing results reported by different laboratories.

## Abstract 2

Several typing methods, with different strengths and weaknesses, are available for studies of *Pneumocystis* pneumonia.

*Pneumocystis* pneumonia (PCP) has been known for many years to be a disease of immunocompromised persons. Before the AIDS epidemic, it had been reported as a cause of death in malnourished infants (*1*). No standardized in vitro propagation system is currently available; consequently, much of the basic biology and epidemiology of *Pneumocystis* spp. remains poorly understood. Advances made over the last 15 years have been largely due to the use of molecular biologic approaches.

For almost 80 years, *Pneumocystis jirovecii* (formerly *carinii*) was considered to be a protozoan. In 1988, DNA studies clearly demonstrated that it was not a single species but a complex group of eukaryotic microorganisms, which were assigned to the kingdom Fungi (*2**–**4*) at the branch point between *Ascomycota* and *Basidiomycota* (*5*).

Many genetic typing methods use DNA sequencing approaches, but others use specific gene probes, single-strand conformation polymorphism (SSCP), or restriction fragment length polymorphisms (RFLP). Genetic typing has shown *Pneumocystis* biodiversity (*6*), environmental reservoirs (*7**,**8*), person-to-person transmission (*9**,**10*), recurrent infections (*11*), subclinical colonization and carriage (*12**–**14*), clinical manifestations (*15*), and sulfa exposure and suspected treatment or prophylaxis failures (*16**–**19*). These studies changed our epidemiologic understanding of PCP, and more studies now suggest collectively that a number of clinical PCP cases are newly acquired rather than activated latent infections (*9**–**12**,**18*).

In laboratories around the world, a number of typing procedures, each with its own strengths and weaknesses, are in use to address the clinical and epidemiologic issues discussed above. In the following sections, we discuss the most common methods, along with examples of how they have been used in molecular epidemiologic studies of PCP.

## Different Typing Methods

A variety of typing methods have been used for *Pneumocystis* genetic analysis, and a large number of gene loci have been examined. We focus on the methods and genes that have been most widely used for molecular epidemiologic analyses or have the greatest potential application.

### DNA Sequence Analysis

Direct DNA sequence analysis is the most common approach currently used for *Pneumocystis* biodiversity and molecular typing studies. Sequence analysis of the thymidylate synthase (TS) and superoxide dismutase (SODA) gene loci, the EPSP synthase domain of the multifunctional arom gene, and the mitochondrial small subunit ribosomal RNA (mt SSU rRNA) locus have been used to distinguish *Pneumocystis* species from diverse mammalian hosts (*6**,**20**,**21*). Because of the generally low sequence divergence among *P. jirovecii* isolates at these loci, they are not highly discriminative for *P. jirovecii* typing. Several additional loci, however, have proved useful for molecular epidemiologic applications (*7**–**19*). These include the internal transcribed spacer (ITS) regions of the nuclear rRNA operon (*9**,**10**,**15*), the mitochondrial large subunit ribosomal RNA locus (mt LSU rRNA) (*7**,**8**,**10*), and the dihydropteroate synthase (DHPS) gene (*10**,**16**–**19*).

#### ITS1 and ITS2

The ITS1 sequence is located on the nuclear rRNA operon between the genes of the 18S rRNA and the 5.8S rRNA, and ITS2 is located between the genes of the 5.8S rRNA and the 26S rRNA (*22*). These noncoding loci are spliced during rRNA synthesis. They show a high level of polymorphism, which has been used for genetic typing applications. The first ITS typing system was developed by Lu et al. (*22*). Using their nomenclature, in which ITS1 alleles are designated with an uppercase letter and ITS2 alleles with a lowercase letter, 15 ITS1 alleles (from A to O) and 14 ITS2 alleles (from a to n) have been described. Based of this amount of DNA polymorphism, a total of 210 *P. jirovecii* types are theoretically possible, with 59 types reported by these authors (*22**,**23*).

A second ITS typing scheme was developed by Tsolaki et al. (*24**–**26*) and is based on nucleotide variation at four positions in the ITS1 and at six positions in the ITS2. According to their nomenclature scheme, ITS1 alleles are designated by using an uppercase letter associated with a numerical subscript, and ITS2 alleles are designated by using a lowercase letter also associated with a numerical subscript. These researchers described six ITS1 alleles and nine ITS2 alleles; these numbers allow for up to 54 potential *P. jirovecii* ITS types, should all possible combinations exist. Laboratories currently using this typing scheme have reported ≈40 different *P. jirovecii* ITS types. The most frequent types are B_1_a_3_ and B_2_a_1_, which have both been identified in one third of all *P. jirovecii* isolates typed to date.

More recently, Nimri et al. (*27*) added to the count of Lee et al. by identifying 12 previously unreported ITS1 alleles and 16 previously unreported ITS2 alleles. In the study by Nimri et al., 36 ITS types were noted in 180 sequences examined from 60 samples. Although the typing methods of Lee et al. and Tsolaki et al. are not strictly identical, a general correspondence between *P. jirovecii* ITS types can be observed with either method. To date, approximately 87 unique ITS types have been identified by the two methods.

#### mt LSU rRNA

The amount of polymorphism reported at this locus is substantially less than that reported for ITS; nevertheless, the variation observed has helped address a number of important epidemiologic questions. The original PCR assay developed for this locus was a single-round PCR that generated a fragment of ≈360 bp (*28*). A nested PCR assay has also been developed (*25*), which has an increased sensitivity and specificity. Recently, this test was used to distinguish subclinical carriage from clinical disease (*14*).

Polymorphism at this locus is routinely reported at two nucleotide positions (85 and 248), showing six unique genotypes. A third variable position has been reported but is rarely seen. Mitochondrial DNA has long been accepted and used as a practical and reproducible tool to evaluate intraspecific variation. Since multiple mitochondria are present in individual organisms but display the same haplotype, mitochondrial loci are more easily detected by PCR than single-copy nuclear genes, which results in generally higher PCR sensitivities.

#### The DHPS Locus

The DHPS locus encodes the key enzyme that is targeted by sulfonamide antimicrobial drugs. Consequently, typing efforts involving this gene have been directed primarily at demonstrating an association between treatment or prophylaxis failures and the specific mutations observed at this locus (*16**–**19*). Polymorphism at this locus has been observed primarily at amino acid positions 55 and 57, where nonsilent mutations have been shown to correlate with sulfonamide exposure and prophylaxis and treatment failure. A nested-PCR assay has been developed for this locus (*29*), with modifications suggested by other investigators (*18*). This assay is highly sensitive and specific for *P. jirovecii* in patients with clinical PCP. In addition to the position 55/57 mutations, nucleotide polymorphism has also been observed at several other sites in the gene (*30*). These mutations have not been shown to result in amino acid substitutions and have not been correlated with adverse clinical outcomes.

### Multitarget PCR-SSCP

A second method for molecular typing *P. jirovecii* involves single-strand confirmation polymorphism (SSCP) analysis (*31**,**32*). The method consists of PCR amplification of four variable regions of *P. jirovecii* (Table), then detecting polymorphism by observing migration pattern variation in gel electrophoresis. The variable regions analyzed include ITS1, the intron of the nuclear 26S rRNA gene (26S), the variable region of the mt LSU rRNA, and the region surrounding an intron of the β-tubulin gene (β-tub).

**Table:** Primer sets for polymerase chain reaction amplification of *Pneumocystis* gene loci commonly used for molecular typing^a^

Gene locus		Primer sequence	Reference
β-tubulin			
Forward		5´ TCA TTA GGT GGT GGA ACG GG 3´	(31)
Reverse		5´ ATC ACC ATA TCC TGG ATC CG 3´	(31)
DHPS (nested)			
1st round	DHPS F1	5´ CCT GGT ATT AAA CCA GTT TTG CC 3´	(28)
	DHPS B_45_	5´ CAA TTT AAT AAA TTT CTT TCC AAA TAG CAT C 3´	(29)
2nd round	DHPS A_HUM_	5´ GCG CCT ACA CAT ATT ATG GCC ATT TTA AAT C 3´	(29)
	DHPS BN	5´ GGA ACT TTC AAC TTG GCA ACC AC 3´	(29)
ITS (nested)			
1st round	1724F	5´ AAG TTG ATC AAA TTT GGT C 3´	(22)
	ITS2R	5´ CTC GGA CGA GGA TCC TCG CC 3´	(22)
2nd round	ITS1F	5´ CGT AGG TGA ACC TGC GGA AAG GAT C 3´	(22)
	ITS2R1	5´ GTT CAG CGG GTG ATC CTG CCT G 3´	(22)
ITS (nested)			
1st round	N18SF	5´GGT CTT CGG ACT GGC AGC 3´	(26)
	N26SRX	5´ TTA CTA AGG GAA TCC TTG TTA 3´	(26)
2nd round	ITSF3	5´ CTG CGG AAG GAT CAT TAG AAA 3´	(24)
	ITS2R3	5´> GAT TTG AGA TTA AAA TTC TTG 3´	(24)
MSG			
Forward	GK242	5´ TAT TTC TTG TAT CTA TGC GCT 3´	(33)
Reverse	GK244	5´ TCC GCG CAA AAA TAA GCA CT 3´	(33)
mt LSU rRNA (nested)			
1st Round	pAZ102-H	5´ GTG TAC GTT GCA AAG TAC TC 3´	(28)
	pAZ102-E	5´ GAT GGC TGT TTC CAA GCC CA 3´	(28)
2nd Round	pAZ102-X	5´ GTG AAA TAC AAA TCG GAC TAG G 3´	(25)
	pAZ102-Y	5´ TCA CTT AAT ATT AAT TGG GGA GC 3´	(25)
Nuclear 26S rRNA			
Forward		5´ GAA GAA ATT CAA CCA AGC 3´	(31)
Reverse		5´ ATT TGG CTA CCT TAA GAG 3´	(31)

^a^DHPS, dihydropteroate synthase; ITS, internal transcribed spacer; MSG, major surface glycoprotein; mt LSU, mitochondrial large subunit.

A variable region amplified from a clinical specimen of a given patient with PCP can generate a simple or complex SSCP pattern. Simple patterns are made of two bands and correspond to a single allele of the genomic region. Complex patterns are made of more than two bands and have been shown to correspond to the superimposition of two simple patterns and the presence of two or, rarely, three alleles of the region (*32*).

**Figure 1:**
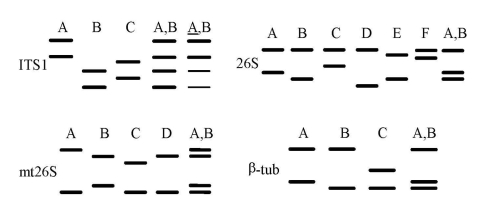
Schematic representation of the single-strand conformation polymorphism (SSCP) patterns of four variable regions used to type *Pneumocystis jirovecii*. Each lane corresponds to a hypothetical sample. All simple patterns with two bands for each region are shown. Each uppercase letter represents a simple SSCP pattern. For each region, the complex SSCP pattern A,B corresponding to the superimposition of simple patterns A and B is represented. The complex ITS1 pattern A,B, is demonstrated, in which pattern A is more abundant than pattern B. Reprinted with permission from reference *32*. Hauser et al. 2001, AIDS:15(4):461–466.

According to their SSCP results, different categories of specimens can be distinguished (*32*). A specimen harboring a single allele at each of the four genomic regions is presumably infected with a single *P. jirovecii* type, and each combination of four simple SSCP patterns defines a type (Figure 1). Studies strongly suggest that a patient harboring two or more alleles of at least one of the genomic regions is coinfected with several *P. jirovecii* types (*31*). Analysis of the alleles and their abundance within the complex patterns allows identification of the coinfecting types in ≈60% of specimens coinfected with two types. Specimens producing at least one complex SSCP pattern made of three simple patterns are presumably infected with at least three types, which cannot be identified. However, the SSCP results of the latter specimens are also informative, as they often allow exclusion of certain specific types. Among 430 specimens from 15 hospitals in five European countries, three to five different simple SSCP patterns could be identified for each genomic region and 43 different *P. jirovecii* types. Thirty percent of the patients were infected with a single *P. jirovecii*, 45% with two types, and 25% with three (*31**,**32*).

### Major Surface Glycoprotein Expression Site Typing

A third, recently reported typing method relies on identifying the number of tandem repeats in the intron of the expression site of the major surface glycoprotein (MSG) of *P. jirovecii* (Table) (*33*). Unlike the other currently available typing methods, which rely on identifying single nucleotide polymorphisms or combinations of such polymorphisms, this method relies on characterizing the size of a region of this intron. Within this region, different *P. jirovecii* isolates have two to six copies of a 10-nt sequence. Typing can be performed by amplifying this region with PCR using primers flanking this region and running the PCR product on a high-resolution acrylamide gel that can separate fragments that differ in size by a few base pairs (*33*).

**Figure 2:**
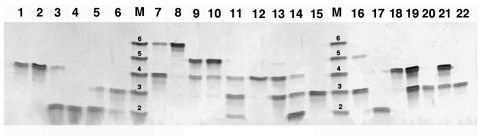
Representative denaturing gel electrophoresis analysis of *Pneumocystis jirovecii* tandem repeats in clinical isolates. Numbers above each lane represent individual isolates. Lane M is a mixture of polymerase chain reaction products from five isolates, of which the number of repeats was 2, 3, 4, 5, and 6 (shown above DNA bands), as determined by sequencing. Reprinted from (*33*) with permission from the University of Chicago Press.

PCR followed by electrophoresis can be used to rapidly determine the number of repeats present in the intron. Because the expression site of the MSG (unlike the MSG itself) is present as a single copy per organism (*34**,**35*), a given strain of *P. jirovecii* will have a unique number of repeats per organism. Infections with more than one strain of *P. jirovecii*, which occur frequently in HIV-infected patients (20%–70% of patients) can be easily detected with this method if the different strains have a different number of repeats, since PCR amplification will result in multiple bands corresponding to the different sizes of the repeats (Figure 2).

The utility of this typing method can be enhanced by sequencing the amplified PCR product because the 10-bp repeats can have one of three sequences (types 1, 2, and 3), which differ from each other by a single nucleotide. Isolates with the same number of repeats can potentially be distinguished from each other by different patterns of repeat types (e.g., three repeats of type 1, 1, 2 are different from three repeats of type 1, 2, 2). However, in isolates with multiple strains of *P. jirovecii*, as determined by quantifying the number of repeats, directly sequencing the PCR product will sequence only the predominant strain. Because adding or deleting 10 bases will shift the homologous sequences by 10, bases will be out of alignment downstream of the shift, making sequencing difficult. Subcloning, followed by sequencing, must be used in these circumstances to determine sequences of minority strains.

**Figure 3:**
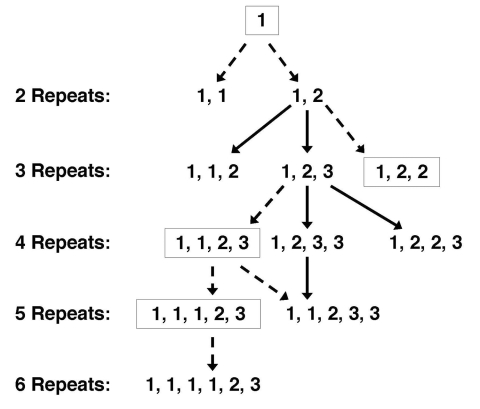
A model for the evolution of tandem repeats in *Pneumocystis jirovecii*, for repeat patterns that have been identified to date. The number of repeats is indicated on the left. The specific pattern of repeats is indicated on the right. The numbers 1, 2, and 3 represent three different repeat types with sequence variation in the first and fourth nucleotides. The repeat patterns that were not identified in this study but are postulated to exist, on the basis of identified patterns, are boxed. Solid arrows indicate potential evolution between isolates that have been identified, and dashed arrows indicate evolution between postulated isolates. Reprinted from (*33*) with permission from the University of Chicago Press.

Analyzing the sequence of repeats also may provide insight into the evolution of *P. jirovecii*. The single base-pair changes seen in repeats likely occurred on a single occasion: since such a mutation appears to be rare (only two unique mutations have been identified) and likely does not confer a biologic advantage to the organism, the same mutation would not likely occur in the same location at different times. Thus, organisms with the 1, 1, 2 pattern of three repeats must have derived from a parental strain with a 1, 2 pattern of two repeats, rather than a 1, 1 pattern of two repeats, both of which have been seen in separate isolates (Figure 3). An analysis of multiple isolates from around the world will potentially provide information about the evolution and spread of *P. jirovecii*. If archival isolates can be identified and examined, we may gain additional information about the evolution of the organism over time.

Because this method was only recently described, it has been used by one group to date. In these limited studies of 147 samples from the United States and Europe, three repeats were most commonly seen, either alone or in combination (*33*). Two and four repeats were seen less frequently, five repeats were seen only in two mixed infections, and six repeats were seen in two isolates, one of which was in a mixed infection with three and four repeats. No pattern related to time of obtaining the sample (1974–2001) or geographic location was identified. Coinfection with more than one strain was identified in 43% of the 147 samples with this method. Additional experience with this typing method, alone and in combination with other methods, is needed to better evaluate its utility in understanding the importance of strain variation and in studying the epidemiology and biologic variability of this organism.

## Assay Stability and Reproducibility

A potential limitation in typing that should be considered in evaluating the various approaches is the shortage of information available on marker stability and assay stability. While these data are generally lacking for most of the commonly used approaches, efforts have been made to evaluate and validate stability over time of multitarget PCR-SSCP (*32*). Specifically, pairs of specimens collected from the same patients during a single PCP episode were analyzed to evaluate the stability of genetic markers. Markers remained stable throughout the 8-week study, which suggests that this method was valid for most clinical applications. Less formal evaluations with direct DNA sequencing show a similar level of stability with the conserved gene loci mt LSU rRNA and DHPS (C.B. Beard, unpub. data). Unexpectedly high rates of ITS variability have been reported in samples collected from the same patient at different times during a single disease episode (*36*). Several possible explanations were proposed, including quantitative changes in the relative abundance of mixed *P. jirovecii* populations, sampling bias, intrinsic instability of the gene locus, and methodologic artifact. Others have observed similar variation patterns at this locus (C.B. Beard, unpub. data), and the explanation is a subject of debate.

## Typing-related Analyses

Two other molecular approaches may address clinical or epidemiologic questions. These applications differ from most typing efforts in that the usual purpose of typing is to evaluate genetic polymorphism, whereas these assays examine organism numbers and viability. A recently developed quantitative PCR assay based on a conserved region of the MSG gene provides a sensitive method for quantifying organism load in oral washes of patients with suspected PCP (*37**,**38*). In the absence of a reliable culture system, PCR-based viability assays directed against mRNA targets have been also been developed (*39**,**40*). Since these applications go beyond the scope of this article, they will not be discussed further; however, they are useful for clinical and molecular epidemiologic studies.

## Best Typing Method

Much consideration has been given to the question of the best genes and best approaches for molecular typing. The answer in most cases will be determined by the typing objective. One consideration is the evolutionary rate of the gene. In most eukaryotic organisms, mitochondrial DNA has been reliable for examining intraspecific variation. In *P. jirovecii*, the mt LSU rRNA locus has generated useful data for addressing specific epidemiologic questions (*7**,**8**,**10*). A greater level of intraspecific variation (*22**–**27*), however, can be detected by using the ITS locus because of its more rapid evolution. Two potential complications associated with the ITS locus are related to assay stability: the specific gene sequence, which includes a polynucleotide stretch of ≈9–12 thymidines that can lead to *Taq* polymerase error during amplification, and the possibility that multiple genotypes occurring in a single isolate could result from two indistinguishable sources, coinfecting *P. jirovecii* strains, or diploid heterozygote organisms in the sample. These concerns do not imply that the locus should not be used but only that these possibilities should be considered when interpreting the data.

The mt LSU rRNA and ITS loci are frequently used because they are not assumed to be under genetic selection and are therefore useful for elucidating molecular evolutionary phenomena that provide the basis for understanding the history of circulating strains. Sometimes, however, typing is employed specifically to determine the existence of genetic selection, such as that induced by exposure to antimicrobial agents (*16**–**19*). Care must be taken in drawing inferences from differences observed at loci that are under genetic selection, since selection can confound inferences concerning strain differences.

DNA sequencing provides the most exhaustive amount of information about any particular DNA fragment, but it is expensive and labor-intensive. Fragment analysis methods such as SSCP are simpler and less expensive, but they rely on having sequence data to characterize the patterns observed. SSCP is also limited in its ability to interpret new genotypes. Both multilocus DNA sequencing and multitarget PCR-SSCP can incorporate information from multiple genetic loci, which allows higher discriminating power to identify strain differences. Low-resolution methods (e.g., RFLP) are best used when the goal is to look only for specific RFLP-defined mutations. Either SSCP or DNA sequencing is suitable for most molecular epidemiologic studies.

## Need To Standardize Reporting

The greatest need in standardization is adopting well-defined sequence types. A good example is the convention used with discussing DHPS mutations, in which the nucleotide or amino acid position is given, along with the specific identity (e.g., Thr > Ala at position 55 and Pro > Ser at position 57 to denote the DHPS double mutant genotype). Using arbitrarily defined numbers or other alphanumeric characters to define genotypes should be avoided, except perhaps for brevity in an article in which observed genotypes are all defined by nucleotide position and identity in a table. The nomenclature systems developed for the ITS locus should be reevaluated; authors’ intentions were good, but they did not account for the variation possible at that locus. A better nomenclature scheme would be to use the specific nucleotide position or variant relative to the original GenBank consensus sequence for that locus, as is typically done when reporting mutations. With the *Pneumocystis* genome project under way and as more genes are cataloged, nomenclature will need to be standardized further, such as with three-letter designations for genes. Within the *Pneumocystis* field, current practice is to use four or more letters to define some loci (e.g., DHFR, DHPS, MTLSU rRNA). Nomenclature standardization, however, should not affect the adoption of standardized typing methods using selected gene targets for specific molecular epidemiologic applications.
